# Comparative evaluation of the influence of television advertisements on children and caries prevalence

**DOI:** 10.3402/gha.v6i0.20066

**Published:** 2013-02-12

**Authors:** Neeta Ghimire, Arathi Rao

**Affiliations:** 1 Department of Pedodontics and Preventive Dentistry, BP Koirala Institute of Health Sciences, Dharan, Nepal; 2 Department of Pedodontics and Preventive Dentistry, Manipal College of Dental Sciences, Mangalore, India

**Keywords:** children, television, advertisement, food purchase, caries, oral health

## Abstract

**Introduction:**

Children watch television during most of their free time. They are exposed to advertisers’ messages and are vulnerable to sophisticated advertisements of foods often detrimental to oral and general health.

**Objectives:**

To evaluate the influence of television advertisements on children, the relationship with oral health and to analyze the content of those advertisements.

**Methodology:**

A questionnaire-based study was performed among 600 schoolchildren of Mangalore, Karnataka, followed by oral examination. Based on the survey, favorite and non-favorite channels and viewing times were analyzed. Advertisements on children’s favorite and non-favorite channels were then viewed, analyzed, and compared.

**Results:**

Higher caries prevalence was found among children who watched television and asked for more food and soft drinks. Cariogenic food advertisements were popular on children’s favorite channels.

**Conclusion:**

Television advertisements may strongly influence children’s food preferences and eating habits, resulting in higher caries prevalence. Advertisements regarding healthy food, oral hygiene maintenance, prevention of diseases such as caries should be given priority for the benefit of the health of children.

Dental caries constitute a significant public health problem worldwide (1). Diet, in particular, sugar-rich food, has always been associated with dental caries (2). Food is a vehicle for nutrient delivery; it provides energy for growth, serves as a structural component, and participates in all metabolic functions of the body. A child’s food choices and dietary habits are influenced by peer pressure, accessibility, and marketing. Food companies sell their products through television advertisement, newspapers, and Internet (3). Marketing methods, such as children’s films, sporting events, film freebies, websites, school books, gifts, on-pack offers, cartoon characters, and film stars, have been used to promote confectionery, high-sugar foods, and junk foods to children (1).

In today’s world, television has become a major part of children’s lives (4). Children watch television during most of their free time. They are exposed to advertisers’ messages and are vulnerable to sophisticated advertising (1). Foods advertised when children are most likely to be watching are high in fat and sugar and low in fiber, with relatively few nutrients (5). Fast foods, high-fat foods, high-sugar foods, and sugar-sweetened beverages are heavily advertised during prime-time programs that target all age groups, including adolescents. Healthy foods, such as fruits, vegetables, whole grains, milk, and low-fat items, are rarely advertised on television. Repeated exposure to high-calorie, low-nutrient foods may increase the craving for these foods. Children who view more commercial television programs make more requests for such items (6).

Companies invest in advertisements on channels watched by children, especially those selling toys and high-sugar food products (1). The relationship between television viewing and dietary behavior has been explored in children and adolescents, but little research has been conducted on the relationship among television advertisement viewing, diet, and dental caries, with reference to increase in food promotion advertisements on television and children’s preference, purchase behavior, and consumption of the advertised product.

The aim of this research was to analyze the influence of television advertisements on children, to see whether there is correlation between television advertisements and children’s oral health, and to examine and compare the nature and content of television advertisements on children’s favorite and non-favorite channels during their popular viewing times.

## Methodology

Ethical committee clearance was taken from Institutional Ethics Committee, Manipal College of Dental Sciences, Mangalore, Karnataka, India. Approval from the school authorities and informed consent from the parents were obtained. Children studying at private schools belonging to similar socioeconomic backgrounds and having television at home were included. Children with special healthcare needs and children/parents who were not willing to participate were excluded. Twelve closed and two open-ended questionnaires (Table 1) were prepared and distributed among students of standard 5, 6, and 7 in their respective schools, followed by oral examinations. All examinations were carried out in a well lit room with additional light using mouth mirrors and standard explorers, by single examiner to avoid interexaminer variations. Dental caries of all the participating children were recorded using DMFT/dmft index.

**Table 1:** Questionnaire

1. Do you watch television? Yes/no
2. If no, what is the reason? There is no TV or cable connection at home Parents do not allow us to watch TV I do not like watching TV Any other reasons
3. When do you watch TV? Morning Afternoon Evening Night Others (9 am–12 noon) (12 noon–4 pm) (4–6 pm) (6–8 pm)
4. How many minutes do you watch TV continuously? <30 min/30–60 min/60–90 min/ > 90 min
5. Which kind of program do you watch regularly? Cartoons/music/movies/serials/any others
6. Which is your most favorite TV channel?
7. Do you like to watch the advertisements in between the programs? Yes/no
8. If no, what is the reason for not watching advertisements? Advertisements are boring Parents do not allow us to watch advertisements I see other channels during advertisements Any other reason
9. Do you buy or ask your parents to buy things that you watch on television? Yes/no/others
10. Items you brought or asked your parents to buy after watching advertisements on TV? Food/soft drinks/tooth paste/tooth brush/dental floss/mouth rinse/dress/cycle/toy/soap/any others
11. What tempted you to buy the above items after watching the television advertisements? Very attractive advertisement with music and color The advertisement has my favorite model I like to buy new things that I get to see in advertisements Television Other reasons
12. How do you select your toothpaste? As advised by the dentist/as advised by TV advertisement/on my own/as decided by parents
13. Have you ever visited dentist for any treatment? Yes/No
14. If yes, can you write for what treatment you had been to a dentist? Filling/cleaning/tooth removal/others

The indices used were DMFT and dmft index. The DMFT index was used for the purpose of assessing the severity of dental caries in permanent dentition.

D=Decayed teeth (only carious cavities were considered as D, Temporary restorations were considered as D.)

M=Missing teeth due to caries

F=Teeth that have been previously filled with permanent restoration.

The dmft index was used for the purpose of assessing the severity of dental caries in deciduous dentition before the age of exfoliation.

d=decayed teeth

m=missing teeth due to caries

f=filled teeth

Each component (d, m, f) was added separately and the total d + m+f=dmft was obtained.

Based on the questionnaire, children’s favorite channels and their viewing time were analyzed. The most commonly viewed three channels and not routinely viewed two channels were viewed during their favorite timing for 4 hours (4–6 pm and 6–8 pm) every 3 days from September 2009 to February 2010. The advertisements during each program were recorded and analyzed for their content and quality.

Food advertisements were grouped as those related to cariogenic and non-cariogenic foods. Those detrimental to oral health, such as chocolate, fast food, soft drinks, biscuits, and energy drinks, were categorized as cariogenic. Fresh food, vegetables, daily items, fresh fruits, and juices were categorized as non-cariogenic. Toys, stationary products, and home items were categorized as non-food items; oral and general health products were categorized as hygiene-related products.

The nature and quality of advertisements during children’s television hours were compared between their favorite and non-favorite channels.

## Statistical analysis

Data were analyzed using Statistical Package for Social Sciences, version 11.5. Frequencies of all the variables were determined. A chi-square test was used for comparison of categorical data. Pearson’s correlation was used to view the relationship between the frequency of television watching and the caries prevalence in children with the level of significance (*P*<0.05).

## Results and observations

**Fig. 1:**
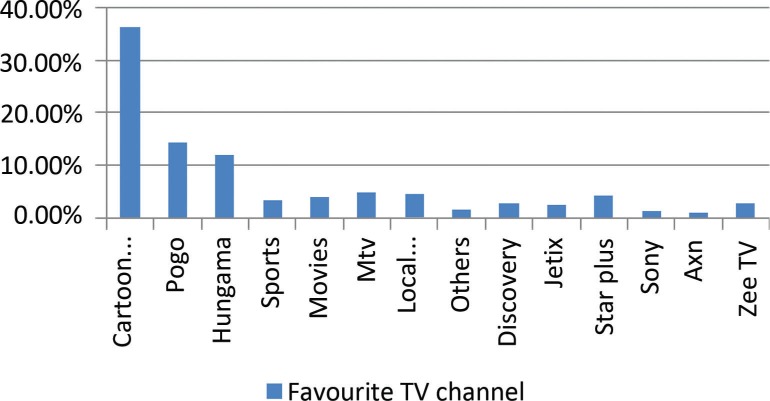
Most preferred channel by children.

Out of 600 children aged 10–13, 59.7% were male and 40.3% were female; 98.8% watched television, whereas 1.2% did not watch television as their parents did not allow it. Of the children, 56.7% watched television between 6 and 8 pm, 33.2% watched between 4 and –6 pm, 5.8% watched between 9 and 12 am, and 5.3% watched between 12 and 4 pm. Of the children, 74.3% watched cartoon channels regularly. Among cartoon channels, the Cartoon Network was found to be the most favorite channel (36.2%), followed by Pogo (14.5%) and Hungama (12%), (Fig. 1). Among 98.8% of the children, 74.2% watched the advertisements between programs, and 25.8% did not watch them. Of the children, 75% were highly influenced by the advertisements and asked their parents to buy advertised products; 66% of the children asked for food and 48.50% asked for soft drinks.

**Fig. 2:**
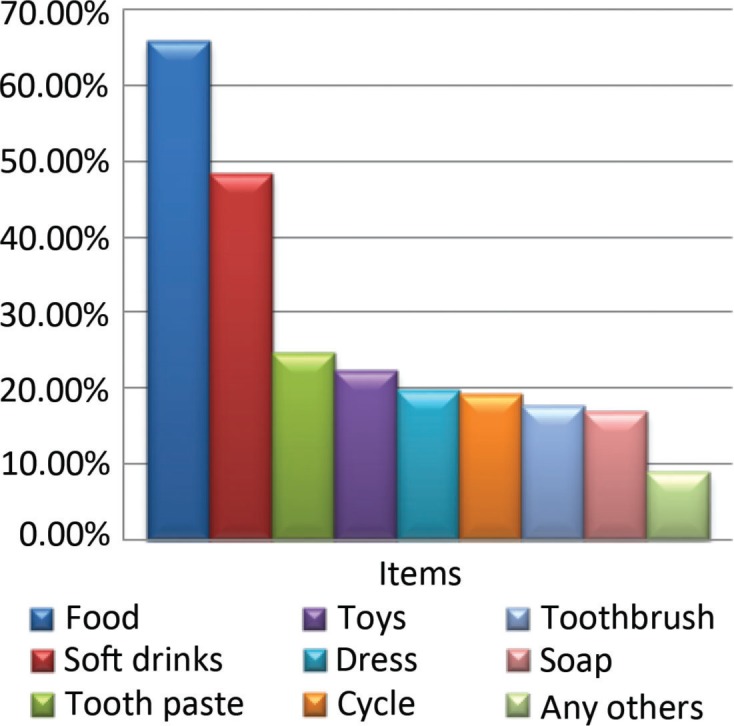
Items children asked after watching advertisements.

Oral health items such as toothbrushes were asked for by 17.70%, toothpaste by 24.70%, mouth rinse by 1%, and 0.70% asked for the purchase of dental floss after watching television advertisements (Fig. 2).

Nearly 28.3% of the children were tempted to buy advertised products due to the presence of their favorite model/movie star in the advertisement, 26.7% wanted to buy just to try new things, and 16.8% were attracted by music and color. About 67.5% (*n*=405) of the children had visited the dentist, of which 34.5% (*n*=207) for restoration, 24.7% (*n*=148) for cleaning, 29.7% (*n*=178) for extraction, and 23.2% (*n*=139) for other reasons.

**Fig. 3:**
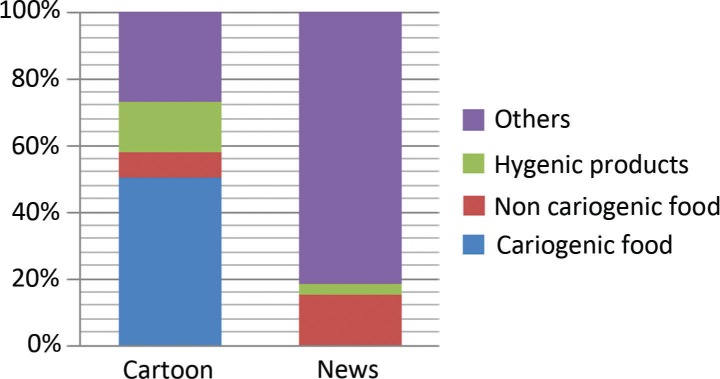
Frequency of advertisements in children’s favorite channel and news channel.

Out of 281 advertisements on children’s favorite channels, 162 were for food products (of which 142 were for cariogenic food), 26.69% (*n*=75) were for non-food products (stationary items, toys), 7.11% (*n*=20) were for oral hygiene products (tooth brushes, tooth paste, and others), and 8.54% (*n*=24) were for other health-related products (Fig. 3). Among these advertisements, 247 had music and songs, 145 had children as role models, 142 had stars/favorite models, and 129 had cartoon characters.

**Fig. 4:**
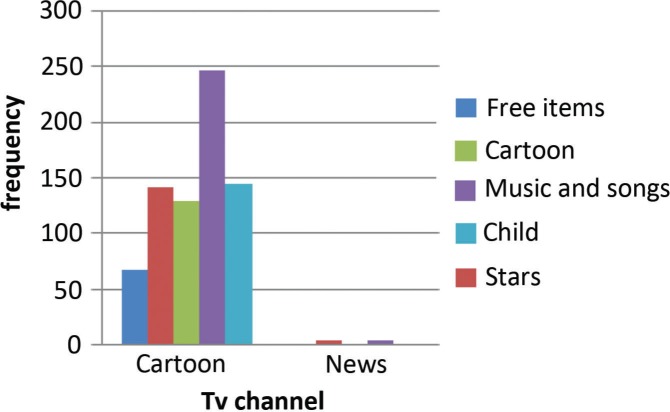
Attractiveness of advertisement in cartoon and news channel.

Among all the advertisements, only 68 offered freebies along with advertised products. On news channels, only 33 advertisements were telecast: 81.81% (*n*=27) for non-food items, 15.16% (*n*=5) for non-cariogenic food items, and 3.03% (*n*=1) was for a hygiene-related product (Fig. 3). None were for cariogenic food product. Music and songs were shown in only five advertisements, four had movie stars/models, and only one had a cartoon character as a role model. Only one advertisement had a freebie (Fig. 4).

Out of the 445 children who watched advertisements in between the programs, 20.4% (*n*=91) had a DMFT score of 1, 18% (*n*=82) had a DMFT score of 2, 4.7% (*n*=21) had a DMFT score of 3, 3.8% (*n*=17) had a DMFT score of 4, and 0.4% (*n*=2) had a DMFT score of 5 (*P*<0.00) (Table 2). Out of the 445 children who watched advertisements in between the programs, 13% (*n*=59) had a dmft score of 1, 15% (*n*=70) had a dmft score of 2, 5% (*n*=24) had a dmft score of 3, 3% (*n*=15) had a dmft score of 4, 4% (*n*=18) had a dmft score of 5, 1% (*n*=5) had a dmft score of 6, and only 0.4% (*n*=2) had a dmft of score 7. The finding was statistically significant (*P*<0.012) (Table 3).

**Table 2:** Correlation between advertisement watching in between the programs and DMFT index

Do you like to watch the advertisements in between the programs?	DMFT permanent								
		
	0	1	2	3	4	5	7	Total	*P*
Yes	232	91	82	21	17	2	0	445	<0.001
No	117	17	14	2	3	1	1	155	
Total	349	108	96	23	20	3	1	600	

**Table 3:** Correlation between advertisement watching in between programs and dmft index

Do you like to watch the advertisements in between the programs?	dmft deciduous									
		
	0	1	2	3	4	5	6	7	Total	*P*
Yes	252	59	70	24	15	18	5	2	445	<0.012
No	117	20	12	3	1	2	0	0	155	
Total	369	79	82	27	16	20	5	2	600	

Among the 434 children who asked their parents to buy things they saw on television, 21% (*n*=92) had a DMFT score of 1, 17% (*n*=77) had a DMFT score of 2, 4.8% (*n*=21) had a DMFT score of 3, 4% (*n*=18) had a DMFT score of 4, and 0.4% (*n*=2) had a DMFT score of 5. Out of the 16 children who sometimes asked their parents to buy things, only 6.25% (*n*=1) had a DMFT score of 1, 18% (*n*=3) had a DMFT score of 2, and 6.25% (*n*=1) had a DMFT score of 4. The level of significance was *P*<0.003 (Table 4). Among 434 children, 13% (*n*=59) had a dmft score of 1, 16% (*n*=70) had a dmft score of 2, 5% (*n*=22) had a dmft score of 3, 3% (*n*=14) had a dmft score of 4, 4% (*n*=18) had a dmft score of 5, 1% (*n*=5) had a dmft score of 6, and only 0.2% (*n*=1) had a dmft score of 1 (*P*<0.002) (Table 5).

**Table 4:** Correlation between purchases of items advertised on television and DMFT score

Do you buy or ask your parents to buy things that you watch on television?	DMFT permanent										
		
	0	1	2	3	4	5	6	7		Total	*P*
Yes		224	92	77	21	18	2	0	0	434	<0.003
No		114	15	16	2	1	1	0	1	150	
Others*		11	1	3	0	1	0	0	0	16	
Total	349	108	96	23	20	3	0	1	600		

* Sometimes.

**Table 5:** Correlation between purchase of items advertised on television and dmft score

Do you buy or ask your parents to buy things that you watch on television?	dmft deciduous									
		
	0	1	2	3	4	5	6	7	Total	*P*
Yes	245	59	70	22	14	18	5	1	434	<0.002
No	110	20	12	3	2	2	0	1	150	
Others*	14	0	0	2	0	0	0	0	16	

* Sometimes.

Among the 396 children who asked for the purchase of advertised food, 21% (*n*=84) had a DMFT score of 1, 18% (*n*=72) had a DMFT score of 2, 4.5% (*n*=18) had a DMFT score of 3, 4.5% (*n*=18) had a DMFT score of 4, and 0.5% (*n*=2) had a DMFT score of 5, with *P*<0.001, which was statistically significant (Table 6). Among the 396 children who asked for the purchase of advertised food, 12% (*n*=50) had a dmft score of 1, 17% (*n*=69) had a dmft score of 2, 5% (*n*=22) had a dmft score of 3, 3% (*n*=14) had a dmft score of 4, 4% (*n*=17) had a dmft score of 5, 1% (*n*=4) had a dmft score of 6, and 0.5% (*n*=2) had a dmft score of 7 (*P*<0.001) (Table 7).

**Table 6:** Correlation between purchase of food advertised on television and DMFT score

	DMFT permanent									
		
Food	0	1	2	3	4	5	6	7	Total	*P*
No	147	24	24	5	2	1	0	1	204	<0.001
Yes	202	84	72	18	18	2	0	0	396	
Total	349	108	96	23	20	3	0	2	600	

**Table 7:** Correlation between purchase of food advertised on television and dmft score

	dmft deciduous									
		
Food	0	1	2	3	4	5	6	7	Total	*P*
No	151	29	13	5	2	3	1	0	204	<0.001
Yes	218	50	69	22	14	17	4	2	396	
Total	369	79	82	27	16	20	5	2	600	

Among the 291 children who asked for the purchase of soft drinks, 20% (*n*=61) had a DMFT score of 1, 19% (*n*=57) had a DMFT score of 2, 4% (*n*=13) had a DMFT score of 3, 4.8% (*n*=14) had a DMFT score of 4, and 0.3% (*n*=1) had a DMFT score of 5 (*P*<0.003) (Table 8). Among the 291 children who asked for soft drinks, 52% (*n*=153) had a dmft score of 0, 13% (*n*=40) had a dmft score of 1, 16% (*n*=49) had a dmft score of 2, 6% (*n*=18) had a dmft score of 3, 3% (*n*=10) had a dmft score of 6, 5% (*n*=16) had a dmft score of 5, 1% (*n*=3) had a dmft score of 6, and 0.6% (*n*=2) had a dmft score of 2 (*P*<0.001) (Table 9).

**Table 8:** Correlation between purchase of soft drinks advertised on television and DMFT score

	DMFT permanent									
		
Soft drink	0	1	2	3	4	5	6	7	Total	*P*
No	204	47	39	10	6	2	0	1	309	<0.003
Yes	145	61	57	13	14	1	0	0	291	
Total	349	108	96	23	20	3	0	1	600	

**Table 9:** Correlation between purchase of soft drinks advertised on television and dmft score

	dmft deciduous									
		
Soft drink	0	1	2	3	4	5	6	7	Total	*P*
No	216	39	33	9	6	4	2	0	309	<0.001
Yes	153	40	49	18	10	16	3	2	291	
Total	369	79	82	27	16	20	5	2	600	

Thus, children who watched television advertisements and asked for food items and soft drinks were found to have more caries and DMFT/dmft index, compared to those who never asked for the purchase of those items, suggesting that watching television advertisements and buying advertised food do have an influence on a child’s oral health.

## Discussion

The commercialization of children’s television programs is one of the several concerns raised by child health professionals, regarding the impact of television on children’s wellbeing (7). Fast food has become a prominent feature of children’s diet throughout the world. Television is one such medium of propagating many food items (8). It is the most efficient and effective promotion tool, especially when the target group is children (9). The current study was done to evaluate the influence of television advertisements on children and the impact on oral health.

Children who watched television advertisements had a greater dmft/DMFT score. Increase of DMFT and dmft score was more among children who asked for the purchase of advertised products (foods, soft drinks). This suggests that advertisements do have an influence on children’s character, behavior, and eating habits, thus resulting in higher caries prevalence. It would be difficult to prove that television advertising has a direct effect on oral health, given the multifactorial nature of dental caries, but a significant correlation between watching television advertisement and dental caries has been shown in the present study.

In this study, most common time for watching television was between 6 and 8 pm (56.7%). On average, children spent 30 min watching television continuously. Hancox, Milne, and Poulton 2004, in their longitudinal study, revealed that males aged 5–15 on average watched television for 2.25 hours per day and females of the same age group watched for 2.14 hours (10). Pine and Nash mentioned that children in the United Kingdom spent an average of 2.5 hours per day watching television (4). Rideout, Roberts, and Foehr 2005 identified that on average, young people spend 6.5 hours a day interacting, often multitasking, with various forms of media, and the majority of time is spent with television and music (11). Most of the children are fond of cartoon channels and watch them regularly (74.3%). The Cartoon Network (39.8%), Pogo (14.5%), and Hungama (12.0%) were the most favorite channels. None of the children viewed news channels.

In this study, 50.53% (*n*=142) advertisements on children’s favorite channels were for cariogenic food and drinks. Among these, most advertisements were for chocolates and drinks. On the news channel, of the few advertisements (*n*=33) that were telecast, 81.81% were related to non-food items, 15.16% to non-cariogenic food, and 3.03% to hygiene-related products. None of the advertisements were related to cariogenic food. Carol Byrd-Bred-Benner revealed that fruits, vegetables, protein-rich foods (i.e. meat, fish, poultry, beans, nuts, and eggs), and dairy products were rarely advertised, whereas foods rich in fats and sweets were advertised frequently, with candy being the most commonly advertised food (12). Maree Scully’s study on the association between commercial television exposure and fast-food consumption among adults found that advertisements for fast food and takeaway occur at a higher rate late in the day, with people viewing television in the evening (6–9 pm) being exposed on average to double the number of fast-food advertisements than people watching television between 7 and 9 am. So, those who watch television during this time will be exposed to larger doses of fast-food advertisements and may engage in the advertised eating behavior (13). Hammond et al. stated that a great number of fast-food advertisements, presenting food of lower nutritional value, is telecast during the early evening hours when children are likely to watch television (14). Rodd and Patel found that 34.8% of the advertisements were related to food and drink products on children’s channels, 95.3% of these being deemed potentially cariogenic or erosive to teeth (1). Likewise, Chestnutt and Ashraf compared the proportion of advertisements promoting foodstuff potentially detrimental to oral health during children’s programs and primetime television. They found that during children’s programs, 73.4% of the advertising time was devoted to foods potentially harmful to teeth as compared to only 18.6% of advertising time during primetime television. Thus, they concluded that children were being bombarded with commercials of sugar-rich products (15, 16).

Mazur et al. found that 71.4% of foods advertised on Polish television are high in fat and sugar; 14.3% of these are soft drinks, and only 14.3% of advertised products can be called healthy (reduced sugar drinks, bio-yogurts). Among adolescents, soft drinks, chips, and snacks are associated with pleasure, being with friends, independence, affordability, and convenience (17). Greenberg et al. found that 91% of heavy viewers of commercial television preferred to purchase advertised products that included toys, food products, and others (4). In this study, 75% of children asked their parents to buy advertised product. Maximum were for food products (66%) followed by softdrinks (48.5%) and toys (22.5%). Chapman, Nicholas, and Supramanium 2006 in their study on content analysis of food advertising on Australian commercial television indicated that viewers are exposed to an average of five food advertisements per hour, with fast food and takeaway (30%) the most advertised food category (13, 18).

In this study, food and soft drinks were asked for very often. Dental products such as toothpaste (24.7%) and toothbrush (17.7%) were asked for by few children. About 0.7% of the children also asked for dental floss. Rise and his group found that the use of dental floss was rare in the samples they surveyed in 22 European countries and Canada. This might suggest the lack of awareness and understanding of this procedure and its value in preventing oral diseases (19, 20). On children’s favorite channel, advertisements for oral hygiene products were related only to toothbrush and toothpaste, and none were related to other dental aids, information on regular visit to the dentist, and other oral hygiene-related instructions. In a study done by Chestnutt and Ashraf, commercials for dental products accounted for 1.2 and 0.3% of advertising time in children’s programs and evening viewing, respectively (15). In this study, only 24.7% of the students purchased toothpaste, as shown in the advertisements.

Another potentially misleading aspect of food advertisement is the characteristics of individuals who eat advertised foods along with animation, pace, fantasy, premium offers (free gifts), attention-grabbing stories, themes of violence, conflict, and achievements (21). Actors/models are usually shown in most of the food advertisements. The actors who eat food tended to be healthy, thin, or with average weight. Obese children never appeared in commercials. These commercials are also misleading because slender, healthy kids were shown gleefully and frequently snacking on high-calorie, low-nutrient foods without consequences typically seen in real life (e.g. obesity, dental caries) (12). In our observation, most of the advertisements showed lean children or adults eating high-calorie foods, and a maximum number of children (28.3%) were tempted to buy the advertised product due to presence of favorite models or actors in the advertisements. Children are fond of trying new things, and this is another reason they are tempted to buy the products. Actors and models were present in a majority of the advertisements in the present study. Advertisements were made more attractive by adding music and songs. Free gifts were another method of attracting children to buy the products. Out of 33 advertisements on news channel (non-favorite channel), actors and models were shown in only four advertisements, music and songs were present in only five advertisements, and only one presented freebies. Promotional activities, such as packaging, pricing, display of the product, and physical organization of the display environment, such as sound and lighting, implemented by marketing experts considerably determine the views and attitudes of the child consumer regarding the product and market places (9).

## Conclusion

A total ban on advertisements would not be practically possible. Advertisements also earn substantial revenue for broadcasting companies. A more reasonable approach would be to limit the number of advertisements that feature potentially unhealthy food and drink products, and also ensure that they ideally carry health warning messages. Advertisements for healthy food, education, and health-related information should be encouraged on children’s favorite channels during their favorite shows. Video clips regarding oral hygiene maintenance, prevention of diseases such as dental caries, and so on featuring sports and movie star with music and dance, can be telecast during their favourite programs. Toothpaste manufacturing companies are doing their part through advertisements. Such projects can be taken up by neutral parties, such as dental associations, either at the local or national level.
